# A robust and statistical analyzed predictive model for drug toxicity using machine learning

**DOI:** 10.1038/s41598-025-02333-z

**Published:** 2025-05-23

**Authors:** Deepak Rawat, Rohit Bajaj, Rachit Manchanda, Ankush Mehta, Prabhu Paramasivam, Suraj Kumar Bhagat, Abinet Gosaye Ayanie

**Affiliations:** 1 Department of Mathematics, Chandigarh University, Mohali, Punjab 140413 India; 2 Department of Computer Sciences, Chandigarh University, Mohali, Punjab 140413 India; 3 Marwadi University Research Center, Department of Mechanical Engineering, Faculty of Engineering & Technology, Marwadi University, Rajkot, 360003 Gujarat India; 4 Department of Research and Innovation, Saveetha School of Engineering, SIMATS, Chennai, 602105 Tamil Nadu India; 5 Marwadi University Research Center, Department of Civil Engineering, Faculty of Engineering & Technology, Marwadi University, Rajkot, 360003 Gujrat India; 6 Department of Mechanical Engineering, Adama Science and Technology University, Adama, 2552 Ethiopia

**Keywords:** Feature selection, Ensembling, Percentage split, Resampling, Saw score, 10-Fold cross validation, Engineering, Mathematics and computing, Toxicology

## Abstract

Over the years, toxicity prediction has been a challenging task. Artificial intelligence and machine learning provide a platform to study toxicity prediction more accurately with a reduced time span. An optimized ensembled model is used to contrast the results of seven machine learning algorithms and three deep learning models with regard to state-of-the-art parameters. In the paper, optimized model is developed that combined eager random forest and sluggish k star techniques. State-of-the-art parameters have been evaluated and compared for three scenarios. In first scenario with original features, in the second scenario using feature selection and resampling technique with the percentage split method, and in the third scenario using feature selection and resampling technique with 10-fold cross-validation. The principal component analysis is performed for feature selection. An optimized ensembled model performs well in comparison to other models in all three scenarios. It achieved an accuracy of 77% in the first scenario, 89% in the second scenario, and 93% in the third scenario. The proposed model shows the performance increase in accuracy by 8% as compared to the top performer Kstar machine learning model and 21% as compared to deep learning model AIPs-DeepEnC-GA which is remarkable. Also there is significant improvement in other important evaluation parameters in comparison to top performing models. Further concept of W-saw score and L-saw is presented for all the scenarios. An optimized ensembled model using feature selection and resampling technique with tenfold cross-validation performs best among all machine learning models in all the scenarios.

## Introduction

The degree to which a medicinal compound is hazardous to living things is known as its toxicity^1^. Toxicology prediction is extremely difficult. Worldwide, numerous medicinal compounds are created each year. Toxicity is related to the amount of chemicals that are inhaled, applied, or injected and can result in death, allergies, or negative consequences on living organisms^2^. A drug’s toxicity can differ from person to person as per their characteristics. Therefore, a dose that is curative for one patient may be poisonous for other^3^. Drugs are necessary for living beings to help with illness, disease diagnosis, or disease prevention^4^. A new medication or chemical molecule must go through a lengthy, expensive process of development. There are two types of chemicals, namely active and inactive ingredients found in every medicine. The term “active ingredients” refers to the substance that constitutes the therapeutic essence of medicine^5^. The other is known as an inactive component, which has no direct therapeutic benefit but is utilized to balance a drug’s potency. Inactive medications are occasionally used to bind, coat, flavor, or even speed up the breakdown of active pharmaceuticals. Therefore, maintaining a balance between active and inactive medications is crucial. The imbalance of active and inactive medications results in toxicity^6^. Thus, predicting drug toxicity is vital. Over the last few decades, toxicity has been a crucial subject of ongoing research^7^. In the past, drug testing was performed on animals followed by human trials but computational intelligence makes it possible to forecast and assess a drug’s toxicity^8^. It is possible to forecast drug toxicity using machine learning approaches^9^. These methods reduce the cost and duration of the evolution process. A critical phase in the machine learning pipeline is FS, where pertinent characteristics are selected in the dataset and excludes redundant attributes^10^. Proper feature selection can shorten training times, prevent overfitting, and enhance model performance. There are numerous ways for selecting features, ranging from straightforward to sophisticated^11^. The best feature subset for a particular machine learning assignment is detected by frequently considering a combination of approaches and experimenting carefully. Additionally to prevent data leakage and estimate performance of model precisely, feature selection must be carried out inside a validated framework^12^. A common dimensionality reduction method in statistics and machine learning is principal component analysis^13^. Its main applications are feature selection and data visualization, with the aim of decreasing a dataset’s dimensionality while retaining as much crucial data as feasible. Principal component Analysis uses linear combinations to produce the main components, and the term “combinations” refers to the linear combinations of the original features^14^. The goal of PCA is to identify a set of orthogonal (i.e., uncorrelated) linear combinations of the initial characteristics that best account for the data’s variation. The original attributes are combined with particular weights or coefficients to create these linear combinations, which are known as the principal components. In brief, PCA diminishes the amplitude of the data by keeping as much information as feasible. The process of dimension reduction is applied by combining the actual features presented as principal components^15^. These combinations are determined by assigning weight to original features that are necessary for structure and reducing the dimension of data. Resampling is a method to change the dataset by addition, deletion, or change of dataset points. This is used to overcome class imbalance and fitting problems in a dataset^16^. Oversampling and undersampling can add biases or lower the quantity of data accessible for training^17^. It must be done with caution. The resampling approach and parameters are persuaded by the dataset, specific challenge, and the desired result. The appropriate model selection, hyperparameter tweaking, and cross-validation must be used in association with resampling to get a balanced and robust machine-learning model^18^. The process of splitting a dataset into subsets for training, testing, and validation is termed as percentage split in machine learning^19^. These subsets are given in terms of the percentage of a dataset. The percentage split selection is based on the size of the dataset and the data accessibility. In a typical percentage split, for training, testing, and validation for instance criteria of 80%, 10%, and 10% can be utilized^20^. Depending upon data size we can build a robust model^21^. However, depending upon the need of a project, the percentage split can be adjusted to get the best machine learning model. To avoid biases, it is important to split data at random or by the use of a method that ensures the best subsets of the entire dataset. K-fold cross-validation is a well-known technique to apply^22^. It helps to estimate the performance on data when a dataset is small. In the technique, the dataset is split into equal size of k-folds. Noted the performance statistics or metrics^23^. Calculate the performance metric(s) average and standard deviation over all K iterations. In comparison to a single train-test split, these statistics offer a more reliable estimation of your model’s performance. The size of our dataset and the available computational resources are only two examples of the many variables that influence the choice of K. K frequently has values between 5 and 10, and 10-fold cross-validation is frequently a suitable place to start. We can experiment with several K values to discover which one gives the most accurate performance estimates for your model^24^. K-fold cross-validation offers a more thorough review than a single train-test split, which can be impacted by the randomness of the split, and aids in evaluating. A number of machines learning algorithms are applied and the performance is evaluated which is quite satisfactory but still the challenges needs to be addressed are overfitting, generalization, dependency on a single factor i.e. accuracy. The presented paper uses k-fold cross validation method to deal with overfitting. Ensembling of lazy and eager is performed to address generalization. Saw scores are composite scores of all performance parameters which strengthens the optimized model.

The main contribution of presented paper is to

- A number of machine learning approaches are already used to improve the performance of the model.
- Optimize and strengthen the model with multidisciplinary domain operational research where W-saw and L-saw are calculated and their respective scores validate the performance of optimized model before deployment.

## Literature review

In this section, different techniques used by researchers in machine learning have been discussed with their findings of the research. Sukumaran et al. created a mongrel method based on particle swarm optimisation and support vector machines to autonomously analyse computed tomography images, offering a high likelihood of detecting the existence of Covid-19-related pneumonia^25^. The model was trained and clarifies the existence of disease in patients that saves time frame for physicians. Sarwar et al. exhibited an ensembled model in deaconing type II diabetes^26^. The authors considered a total of 15 models but used five main approaches. To achieve the desired results they employed matrix laboratory and the weka tool. The voting technique is used in ensembling the classifiers. A medical dataset of 400 people around the globe is considered during the research. Verma et al. provided an analysis of machine learning methods, both supervised and unsupervised, for identifying incredulous behaviour^27^. The authors studied the behavior of a single person in a crowd with artificial intelligence techniques. Bojamma et al. studied the importance of plant identification in balancing the nature and saving the geodiversity of a zone^28^. The authors assessed the condition to explore latest approaches for systematic identifications of flora. The combined efforts of artificial intelligence and botanists are important to robotize the complete method of recognition of plants considering leaves as crucial characteristics that help identify between different plants. Shidnal et al. studied about lack of nutrients in a paddy crop. They used neural network to categorise the shortcomings using tensor flow^29^. Clustering technique k means is applied to build clusters^30^. The authors estimated state of deficiencies on a measurable basis. A rule-based matrix is also used to estimate cropland’s yield. Table 1 represents the literature based on the algorithm used in the study.

**Table 1:** Literature based on the algorithms studied.

Author	Algorithms											
	SVM	RF	KNN	NB	NN	DT	ANN	J48	MLP	BN	LR	Other
^31^	Y	Y	Y	Y	Y	N	N	N	N	N	N	N
^32^	Y	Y	Y	Y	N	N	N	N	N	N	N	N
^33^	Y	Y	Y	Y	N	Y	Y	N	N	N	N	N
^34^	Y	Y	N	N	N	N	N	Y	N	N	N	Y
^35^	Y	Y	N	Y	N	N	N	Y	N	N	N	N
^36^	Y	Y	Y	Y	N	N	Y	Y	N	N	N	N
^37^	N	N	Y	N	N	N	N	N	N	N	N	Y
^38^	Y	Y	N	N	N	N	N	N	N	N	N	Y
^39^	N	Y	N	N	Y	Y	N	N	N	N	N	Y
^40^	N	Y	N	N	Y	Y	N	N	N	N	N	Y
^41^	Y	Y	N	N	Y	N	N	Y	N	Y	Y	Y

Tables 2 and 3 present the study of evaluation parameters and approaches used in different research respectively.

**Table 2:** Literature based on the evaluation parameters.

Author	Evaluation Parameters									
	Q	SE	SP	AUC	F-1	R	R^2^	MAE	RMSE	Others
^31^	Y	Y	Y	N	N	N	N	N	N	N
^32^	Y	Y	Y	Y	N	N	N	N	N	N
^33^	Y	Y	Y	N	N	N	N	N	N	Y
^34^	Y	Y	Y	Y	N	N	Y	N	N	Y
^35^	Y	N	N	N	N	N	N	N	N	Y
^36^	Y	Y	Y	Y	N	N	N	N	N	N
^37^	N	N	N	N	N	Y	Y	Y	Y	N
^38^	Y	Y	Y	Y	N	Y	Y	N	N	N
^39^	Y	N	N	N	N	Y	Y	N	Y	N
^40^	Y	N	N	N	N	Y	Y	N	Y	N
^41^	Y	N	N	Y	Y	N	N	N	N	Y

**Table 3:** Literature based on the approaches used.

Author	Approaches						
	Classification	Regression	Ensemble	Feature Selection	Class Balancing	Hybrid	Others
^31^	Y	N	Y	N	N	N	N
^32^	Y	N	Y	N	N	N	Y
^33^	Y	N	N	N	N	N	N
^34^	Y	N	Y	Y	Y	N	N
^35^	Y	N	N	N	Y	N	N
^36^	Y	N	N	Y	N	N	N
^37^	Y	N	N	N	N	N	Y
^38^	Y	Y	Y	Y	N	N	Y
^39^	N	Y	N	Y	N	N	N
^40^	N	Y	N	Y	N	N	N
^41^	Y	N	Y	Y	Y	N	N

Renowned researchers are applying machine learning algorithms to understand highly complex problems. There is always a need for pre-processing to better understand complex data. New techniques are needed for pre-processing methods like feature selection and clustering as well. Prominent work is done by the researchers in the field, now ensembling in the feature selection is necessary to make a robust model. Gini index, principal component analysis, recursive feature selection, fisher filtering, Lasso regression, correlation attribute evaluator, and many more feature selection methods are used by researchers. Now researchers are optimizing their prominent work toward hybrid or ensembling of algorithms.

The paper is structured as:

I) Section “Proposed Methodology” depicts methodology adopted.
II) Section “Results and discussions” presents detailed discussions about the dataset and results achieved in all the three scenarios described.
III) Section “Conclusion” presents conclusion and future scope.

## Proposed methodology

**Fig. 1:**
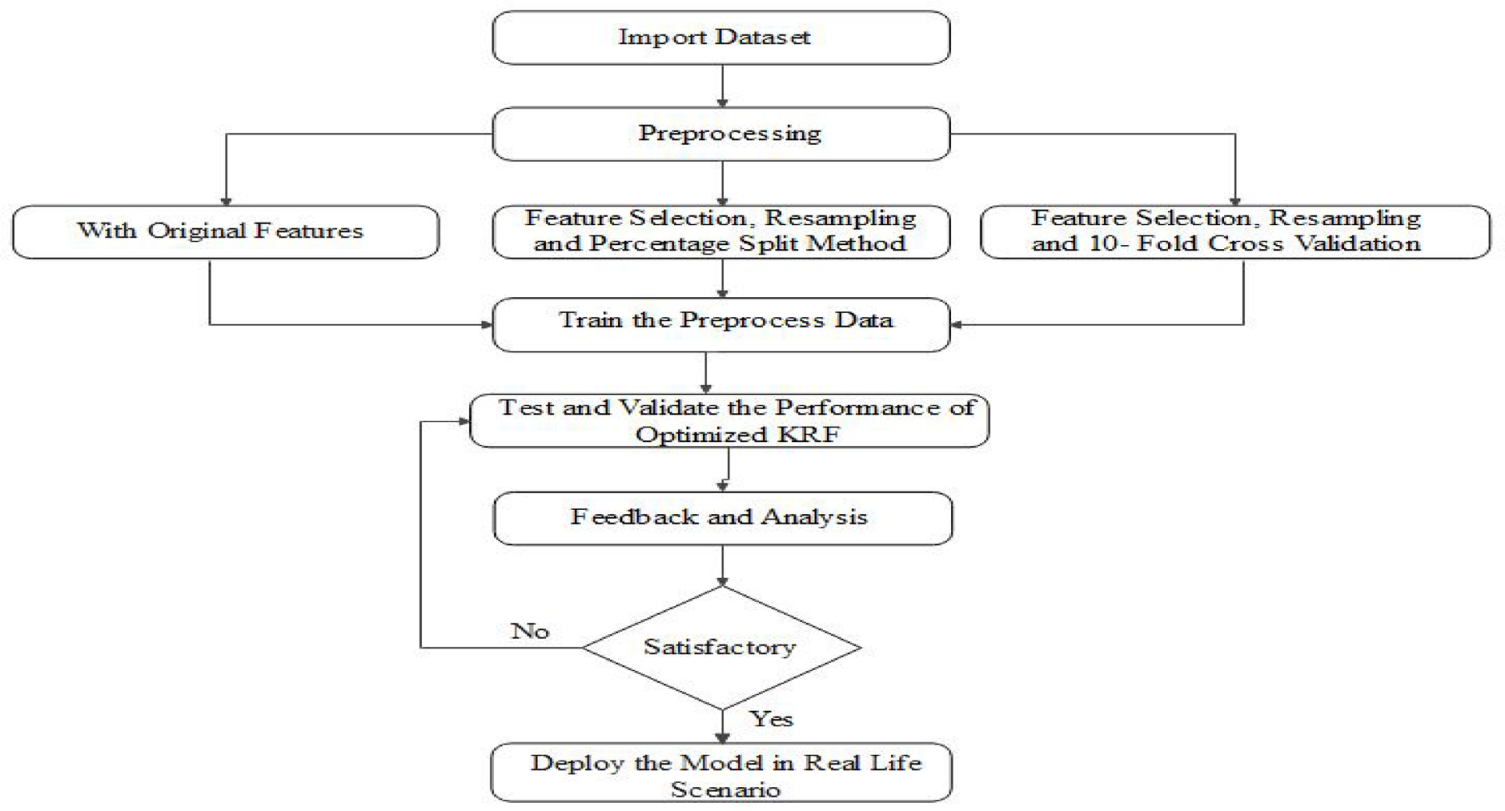
Methodology.

In the research, seven computer-aided machine learning models are evaluated and the performance is compared. Gaussian Process (GP), Linear Regression (LR), Sequential monitoring optimization (SMO), Kstar, Bagging, Decision Tree (DT) and Random Forest (RF) are taken into consideration to predict toxicity. Through ensemble of the Random Forest and Kstar algorithms, we were able to develop an optimized ensembled model (OEKRF). Three scenarios are introduced for the preprocessing and training of data. Seven machine learning algorithms and optimized KRF are evaluated and compared in all three scenarios. Results are compared in aspects state of art parameters. Further to strengthen the model, W-saw and L-saw scores are also evaluated, and the framework of a robust model is deployed. Figure 1 represents methodology proposed for the model.

Pseudocode is also presented below to elaborate the process in detail:
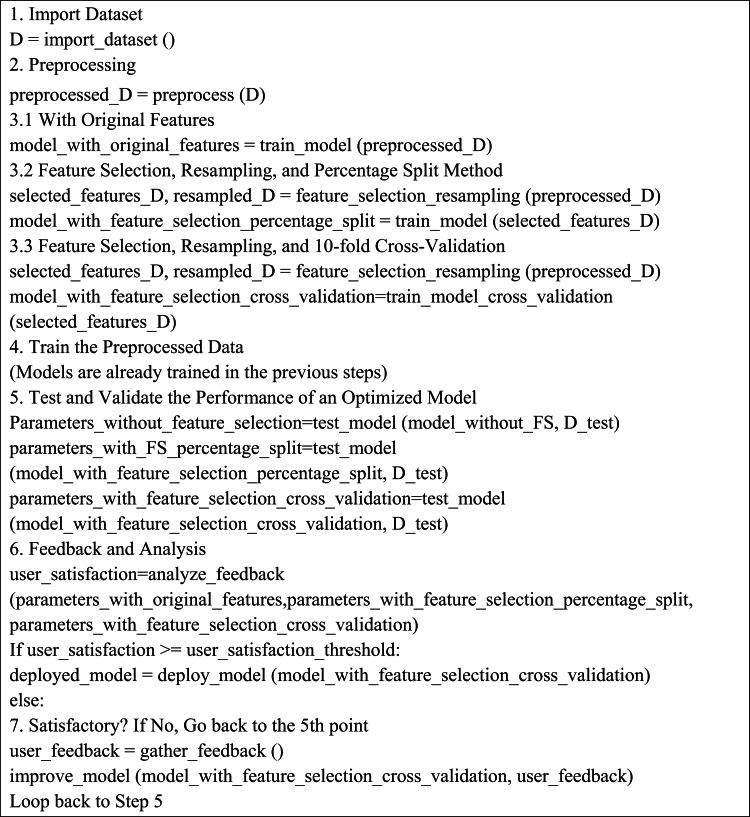


## Results and discussions

This section is divided into two subsections. First subsection presents the dataset description in which different attributes of dataset are described and the second subsection depicts the result analysis with discussions.

### Dataset description

The toxicity dataset that is used in an implementation is obtained from UC Irvine ML depository. There are 546 instances in the dataset, and there are eight predictive attributes. The attributes are listed in Table 4 and a detailed description is also presented.

**Table 4:** Attribute description.

Attributes	Description of Attributes
T.P.S.A.-(Tot)	Area of the topological polar surface
S.A.acc.	Acceptors of surface area
H (050)	Count of hydrogen atoms
M-LOG-P	Moriguchi values of LOG P
RDCHI	Demonstrates the topological index
GAT S1p	Symbolizes the polarisability of molecules
N.Nitrogen	Count of atoms of nitrogen
C (040)	Count of atoms of carbon

Table 5 shows some tuples of dataset which are representative of the entire dataset.

**Table 5:** Tuples of dataset.

TPSA(Tot)	SAacc	H-050	MLOGP	RDCHI	GATS1p	nN	C-040
35.53	47.145	0	4.579	3.875	1.124	0	1
17.07	25.145	0	0.202	1.225	1.66	0	0
34.14	50.29	0	0.076	1.654	1.493	0	0
67.51	103.973	1	3.204	3.344	0.938	0	1
49.33	85.839	2	1.06	2.272	1.007	1	1
122.22	155.543	2	1.406	3.511	1.456	5	2

In the paper, the principal component analysis technique is employed to procure prime combination of the features. Principal component analysis is performed in conjunction with ranker research method. Dimensionality reduction is done by choosing eigen vectors to account for some percentage of the variance in the original data. Five new combinations (NC) have been introduced for the optimized toxicity prediction model. Table 6 presents the description of new features as per the technique.

**Table 6:** New combinations by principal component analysis.

NC1	−0.496SAacc-0.492 TPSA(Tot)−0.439 H-050-0.374nN + 0.282 MLOGP…
NC2	−0.658 MLOGP-0.613RDCHI + 0.411GATS1p-0.085SAacc-0.081nN…
NC3	−0.842GATS1p-0.374RDCHI + 0.302 H-050 + 0.174nN-0.163 MLOGP…
NC4	0.873nN-0.357 H-050-0.309SAacc + 0.096GATS1p-0.07RDCHI…
NC5	0.69 H-050-0.58 TPSA(Tot) + 0.278 MLOGP + 0.242GATS1p + 0.207nN…

The correlation among the various combinations is shown in Table 7.

**Table 7:** Coefficient of correlation among different combinations.

	NC1	NC2	NC3	NC4	NC5
NC1	1	0.02	0.03	−0.21	0.12
NC2	0.02	1	−0.06	−0.01	0.02
NC3	0.03	−0.06	1	−0.01	−0.04
NC4	−0.21	−0.01	−0.01	1	0.1
NC5	0.12	0.02	−0.04	0.1	1

**Fig. 2:**
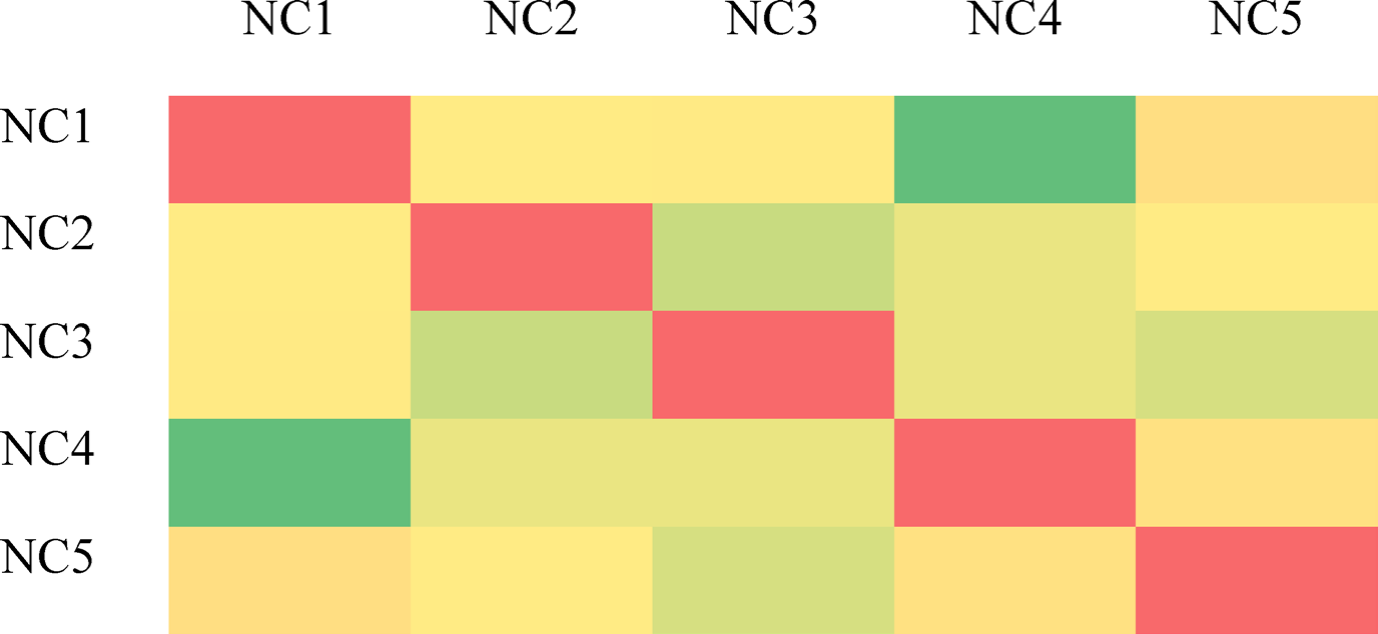
Heat map.

Heat map is a technique used for data visualization which represents numerical values in the dataset by using different color combinations. It depicts correlation coefficients by color gradients. In Fig. 2, red color depicts the highest value of correlation coefficient, yellow color shows the mediate values and green color shows the lowest value of correlation coefficient.

**Fig. 3:**
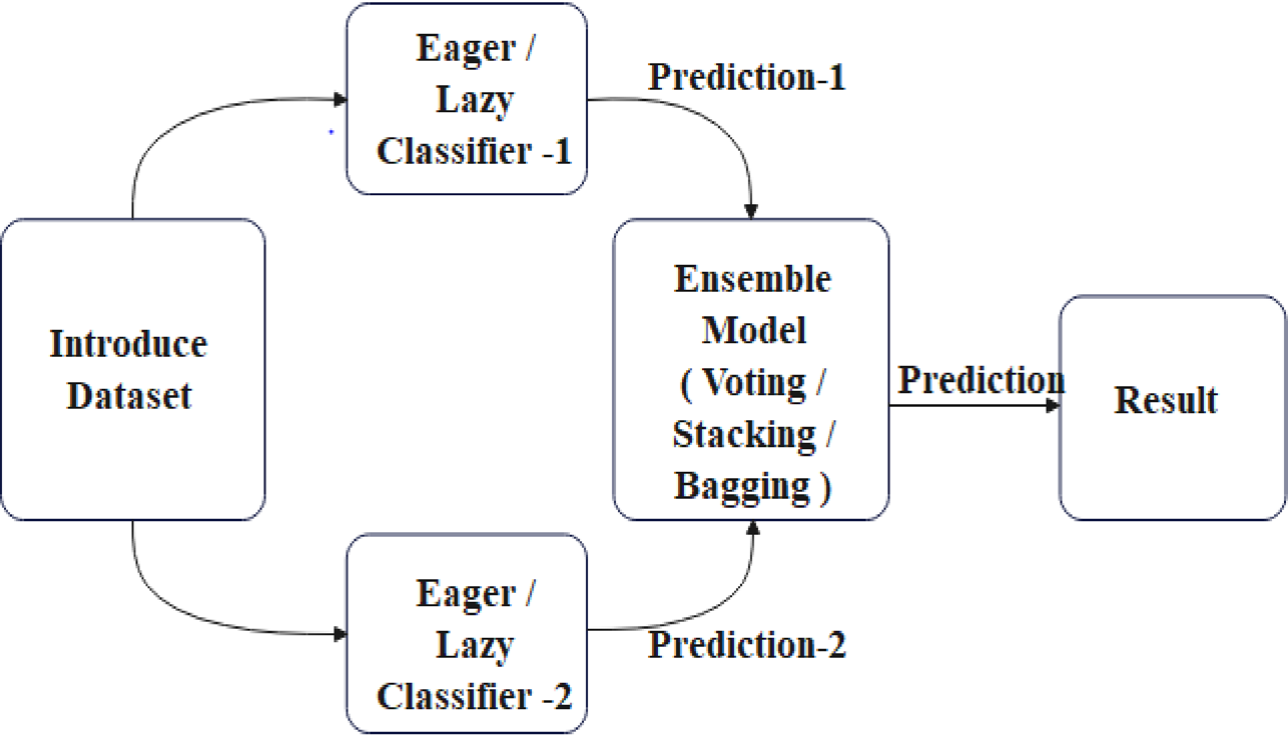
Ensembled Model.

Through ensemble of the Random Forest and Kstar algorithms, we were able to create a better regression model (OEKRF). Figure 3 represents the methodology for ensembled model and Classifier − 1 and classifier- 2 are applying a lazy and eager algorithm for prediction. Further ensembling is performed using different algorithms.

**Figure:**
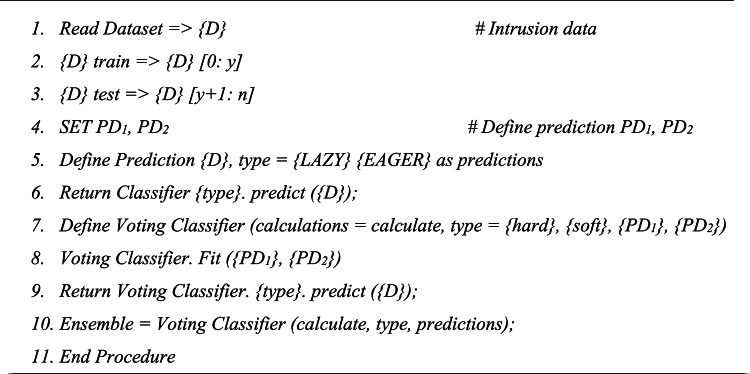
Algorithm: Prediction and Ensembling

### Results and discussions

Three different scenarios have been considered for the evaluation and comparison of seven machine-learning algorithms and optimized KRF as follows:

I) Evaluation and comparison with original features.
II) Evaluation and comparison with feature selection, resampling, and percentage split method.
III) Evaluation and comparison with feature selection, resampling, and 10-fold cross-validation method.

Coefficient of correlation is denoted by R value. It represents how much one variable is correlated to another variable. The value may be positive or negative. It varies from − 1 to 1. The coefficient of determination (COD), also referred to as the R2 score, is used to evaluate how effective a regression model is. The degree of change in the output dependent characteristic can be predicted from the input independent variables. When the COD score is 1, the data were correctly predicted by the regression. It ranges from 0 to 1. MAE and RMSE is a statistical indicator used to assess the efficacy of a machine learning algorithm on a particular dataset. It contrasts the variations between actual data and predictions while outlining the model evaluation error. Q represents the accuracy of the models in percentage. State of art parameters is presented in Table 8 for a scenario I i.e. with original features. An optimized ensembled KRF is evaluated best with the R value as 0.9, COD value as 0.81, MAE value as 0.23, and RMSE value as 0.3. Accuracy is also best for an ensembled model and the observed value is 77% in scenario I.

**Table 8:** State of art parameters in scenario I.

Sr. No.	Classifier	*R* I	COD I	MAE I	RMSE I	Q I
1	GP	0.54	0.29	0.47	0.5	53%
2	LR	0.59	0.35	0.42	0.5	58%
3	SMO	0.59	0.35	0.43	0.5	57%
4	Kstar	0.58	0.34	0.36	0.5	64%
5	Bagging	0.61	0.37	0.4	0.5	60%
6	DT	0.37	0.14	0.46	0.6	54%
7	RF	0.63	0.4	0.37	0.5	63%
8	OEKRF	0.90	0.81	0.23	0.3	77%

State of art parameters is depicted in Table 9 for scenario II i.e. with feature selection, resampling, and percentage split method. Optimized ensembled KRF has performed well again in comparison to other machine learning algorithms with R value as 0.91, COD value as 0.83, MAE value as 0.11, and RMSE value as 0.28. It performed well in terms of achieving an accuracy of 89% in scenario II.

**Table 9:** State of art parameters in scenario II.

Sr. No.	Classifier	*R* II	COD II	MAE II	RMSE II	Q II
1	GP	0.58	0.34	0.45	0.48	55%
2	LR	0.62	0.38	0.4	0.47	60%
3	SMO	0.61	0.37	0.36	0.43	64%
4	Kstar	0.73	0.53	0.19	0.36	81%
5	Bagging	0.69	0.48	0.36	0.42	64%
6	DT	0.77	0.59	0.46	0.46	54%
7	RF	0.82	0.67	0.24	0.42	76%
8	OEKRF	0.91	0.83	0.11	0.28	89%

State of art parameters is compared in Table 10 for scenario III i.e. with feature selection, resampling, and 10-fold cross-validation method. The optimized ensembled model in scenario III outperforms all other models by achieving an accuracy of 93%, R value as 0.93, COD value as 0.86, MAE value as 0.07 and RMSE value as 0.25.

**Table 10:** State of art parameters in scenario III.

Sr. No.	Classifier	*R* III	COD III	MAE III	RMSE III	Q III
1	GP	0.63	0.40	0.43	0.48	57%
2	LR	0.68	0.46	0.35	0.44	65%
3	SMO	0.63	0.40	0.33	0.42	67%
4	Kstar	0.82	0.67	0.15	0.34	85%
5	Bagging	0.73	0.53	0.32	0.39	68%
6	DT	0.79	0.62	0.42	0.43	58%
7	RF	0.87	0.76	0.19	0.37	81%
8	OEKRF	0.93	0.86	0.07	0.25	93%

**Fig. 4:**
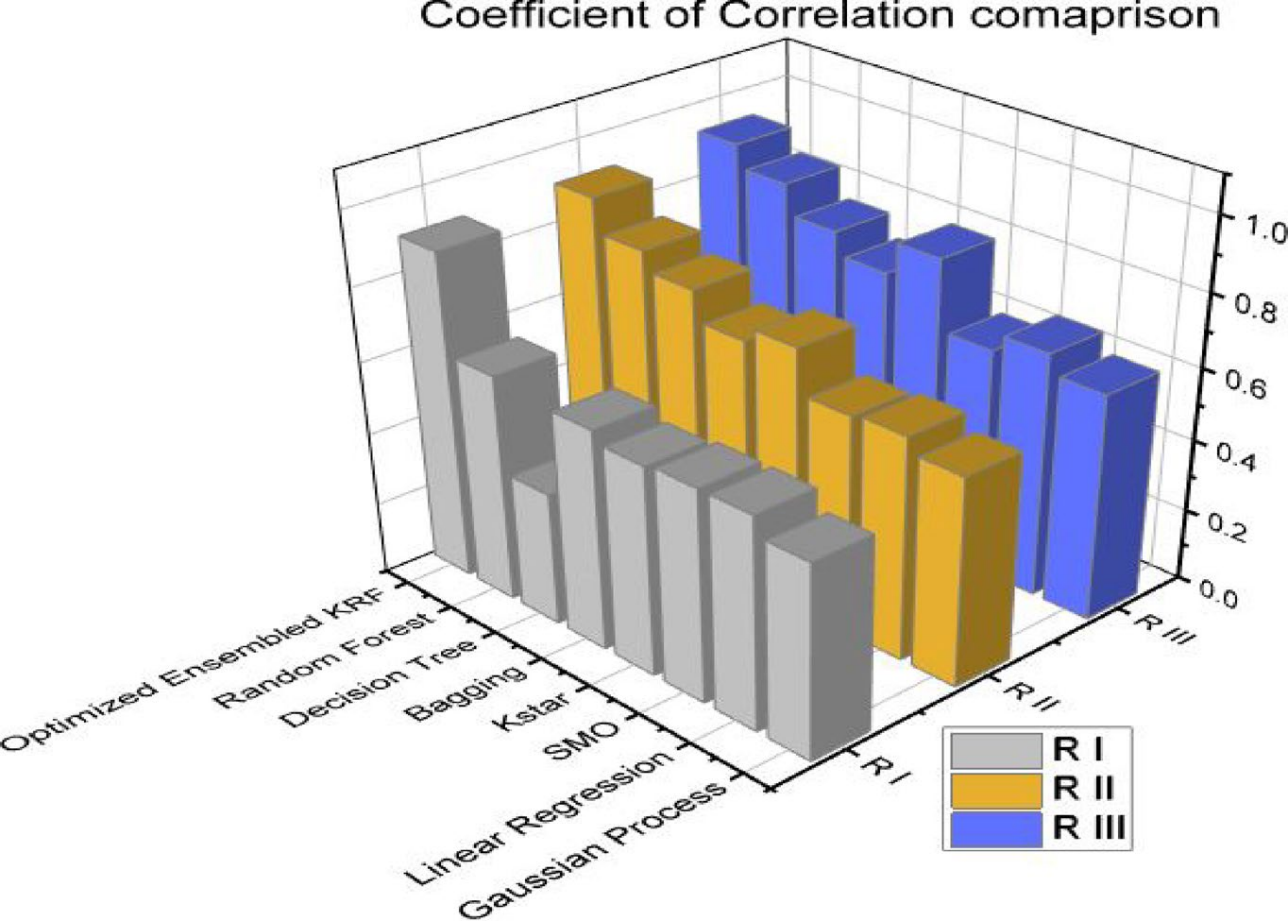
Coefficient of correlation comparison in all three scenarios.

**Fig. 5:**
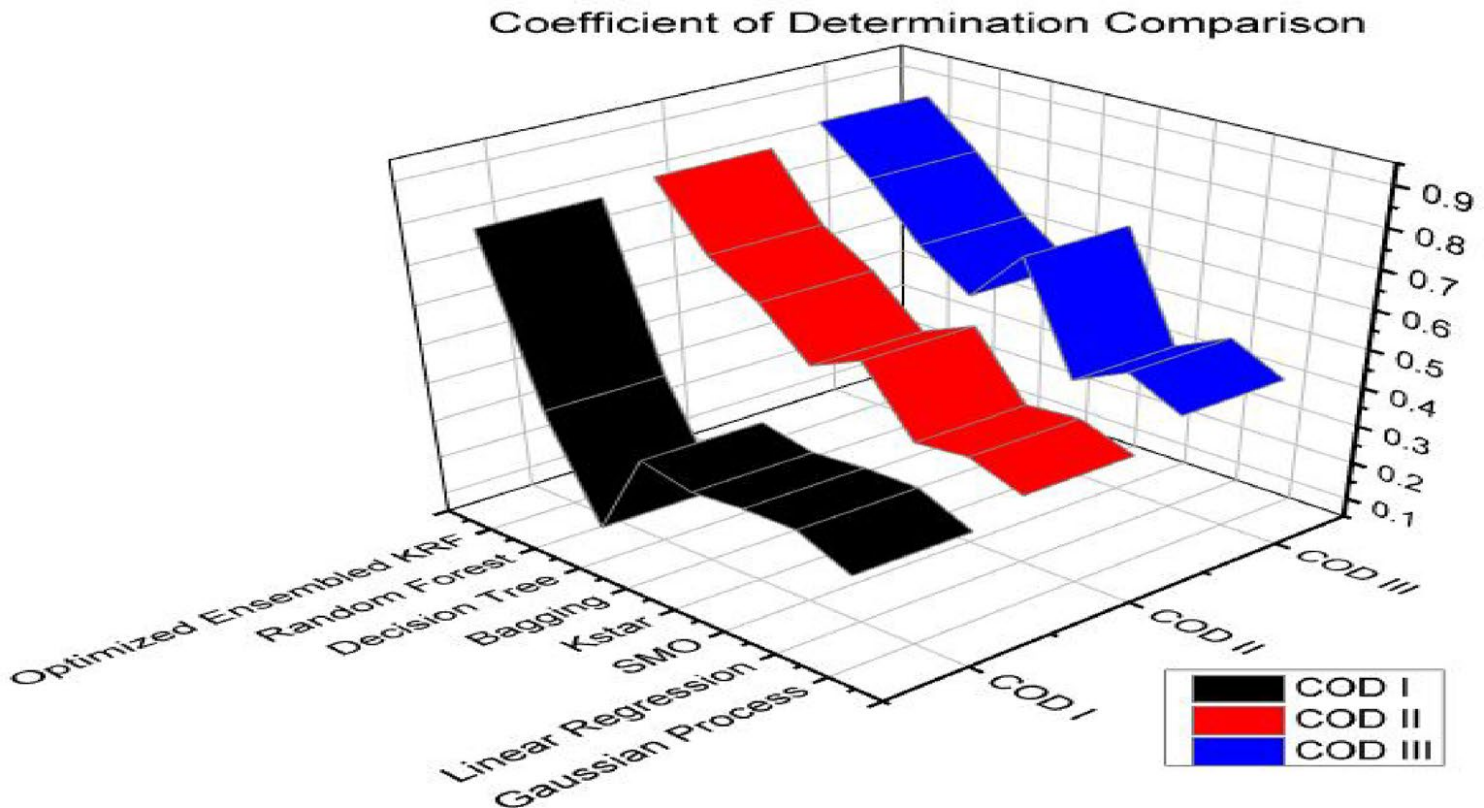
Coefficient of determination comparison in all three scenarios.

**Fig. 6:**
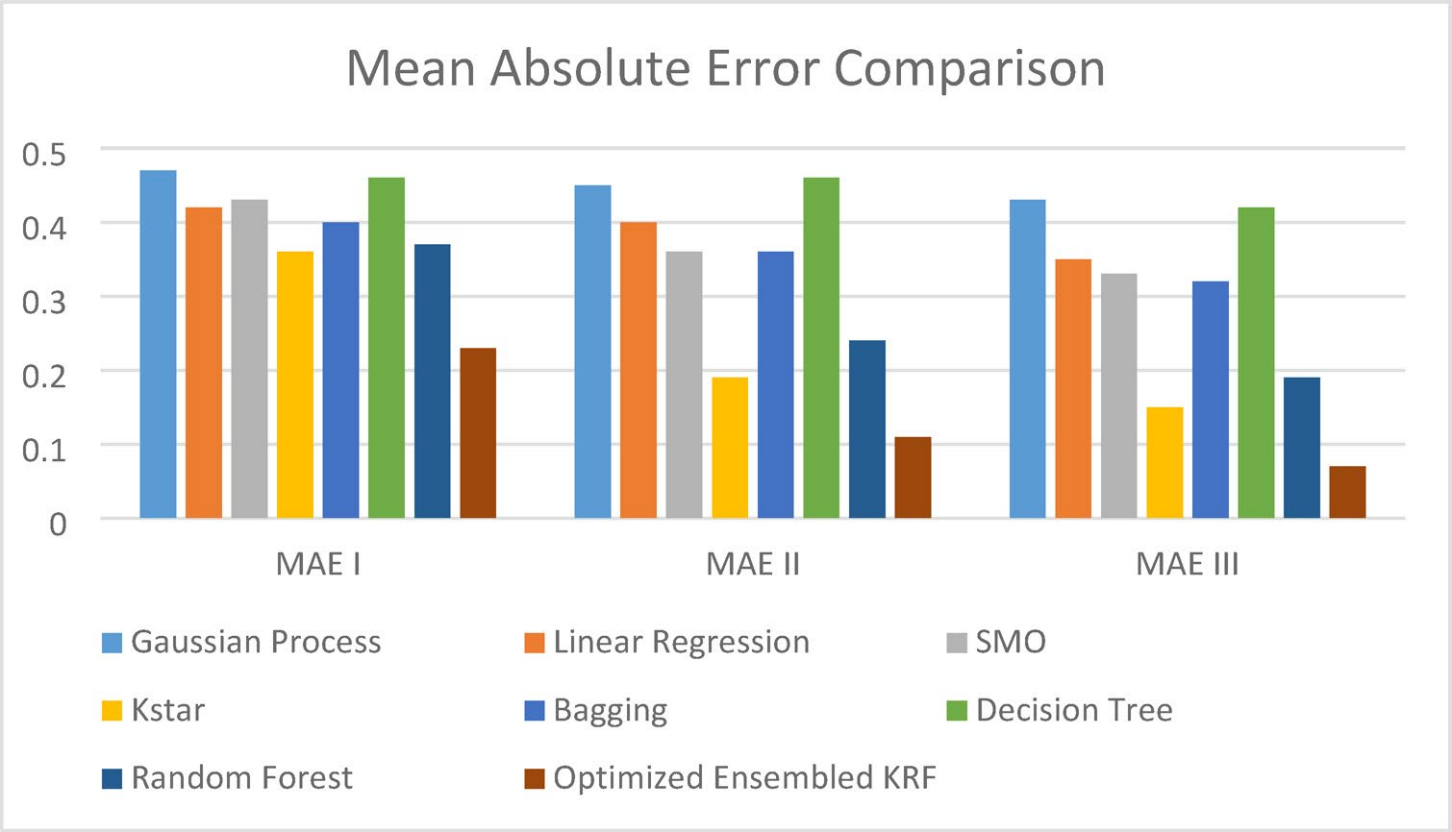
Mean absolute error comparison in all three scenarios.

**Fig. 7:**
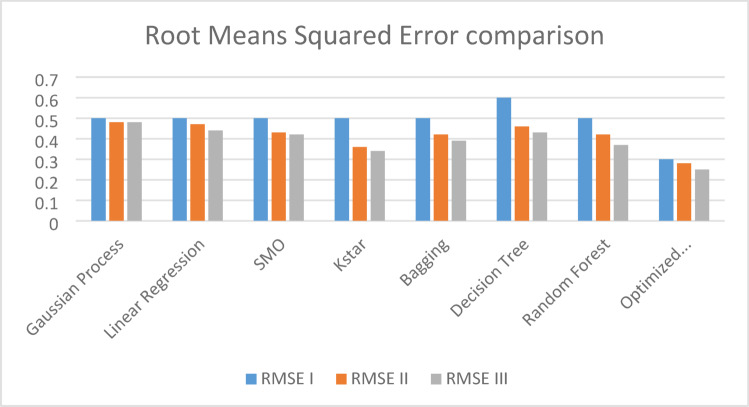
Root mean squared error comparison in all three scenarios.

Figures 4 and 5 present comparison of coefficient of correlation (R) and coefficient of determination (COD) in all the three scenarios respectively. Figures 6 and 7 depict the MAE and RMSE in all the three scenarios respectively.

**Fig. 8:**
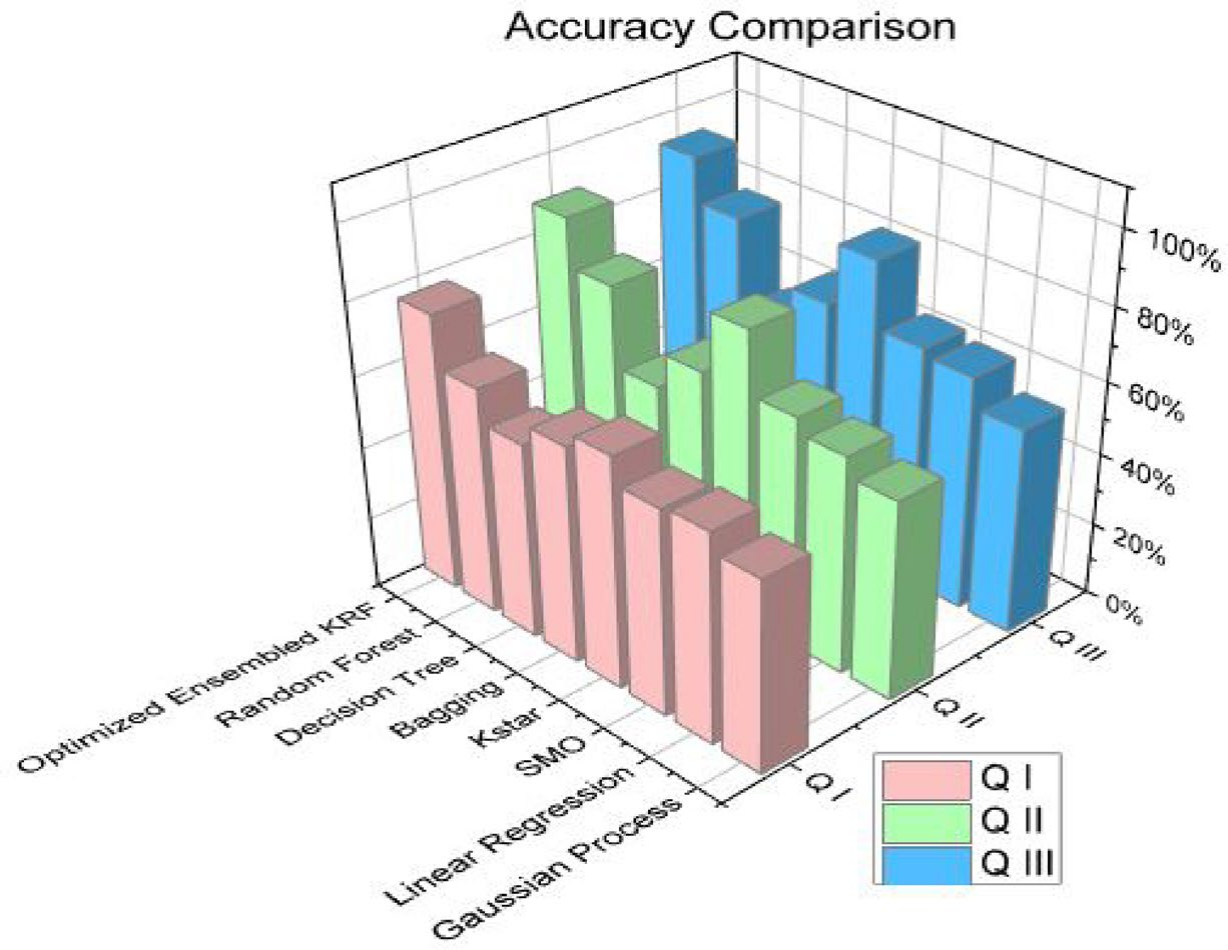
Accuracy comparison in all three scenarios.

The optimized ensembled toxicity prediction model KRF performs well in all the scenarios in comparison to seven machine learning algorithms. When the optimized model is compared in all three scenarios, it performed best in scenario III. Accuracy comparison is shown separately in Table 11; Fig. 8 to present the performance of all models together. The highest accuracy is achieved by optimized KRF in scenario III as 93%. For scenario I and scenario II, the accuracy achieved by the optimized model is 77% and 89% respectively.

**Table 11:** Accuracy comparison for three scenarios.

		Accuracy		
Sr. No.	Classifier	Q I	Q II	Q III
1	GP	53%	55%	57%
2	LR	58%	60%	65%
3	SMO	57%	64%	67%
4	Kstar	64%	81%	85%
5	Bagging	60%	64%	68%
6	DT	54%	54%	58%
7	RF	63%	76%	81%
8	OEKRF	77%	89%	93%

**Fig. 9:**
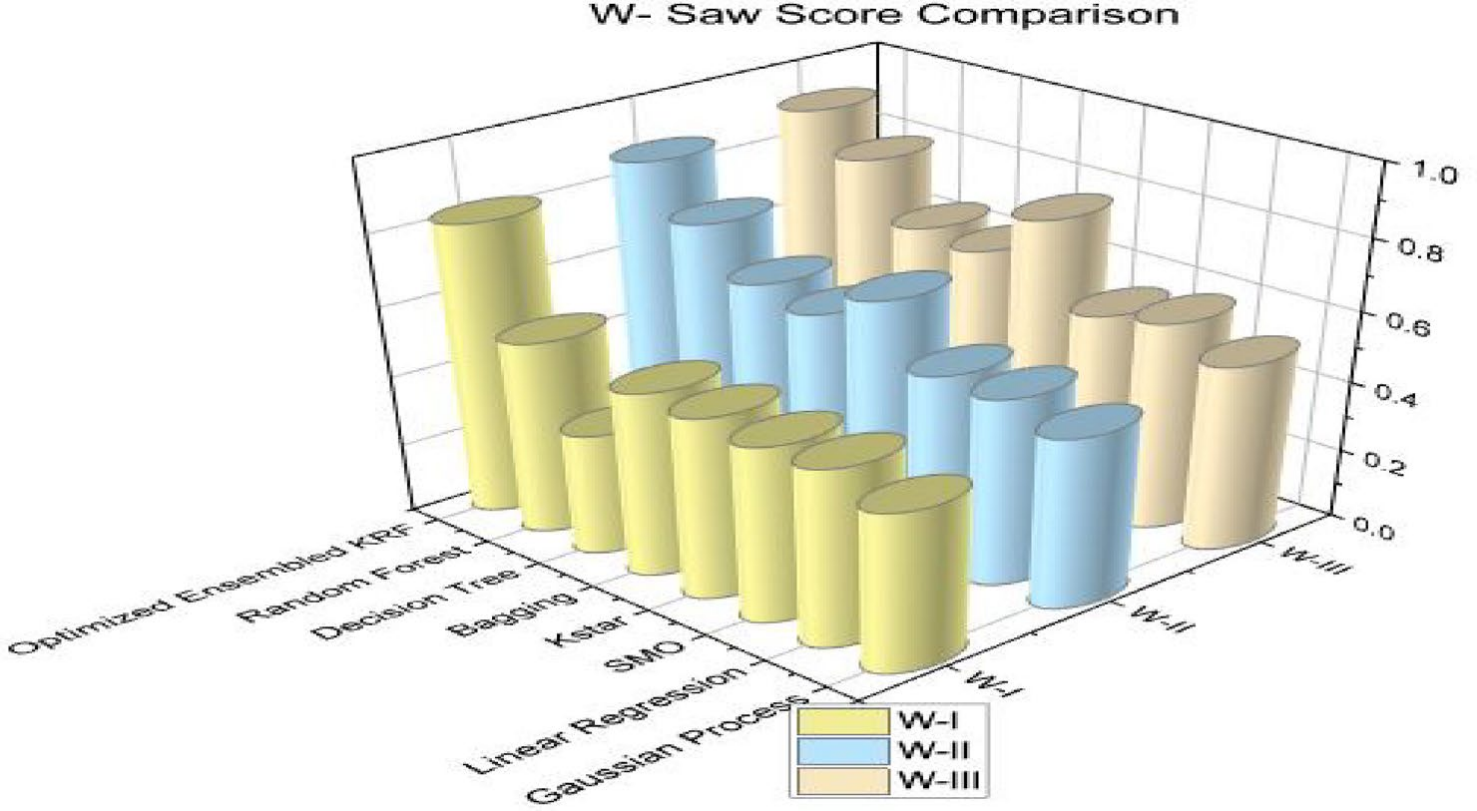
W-saw comparison in all three scenarios.

**Fig. 10:**
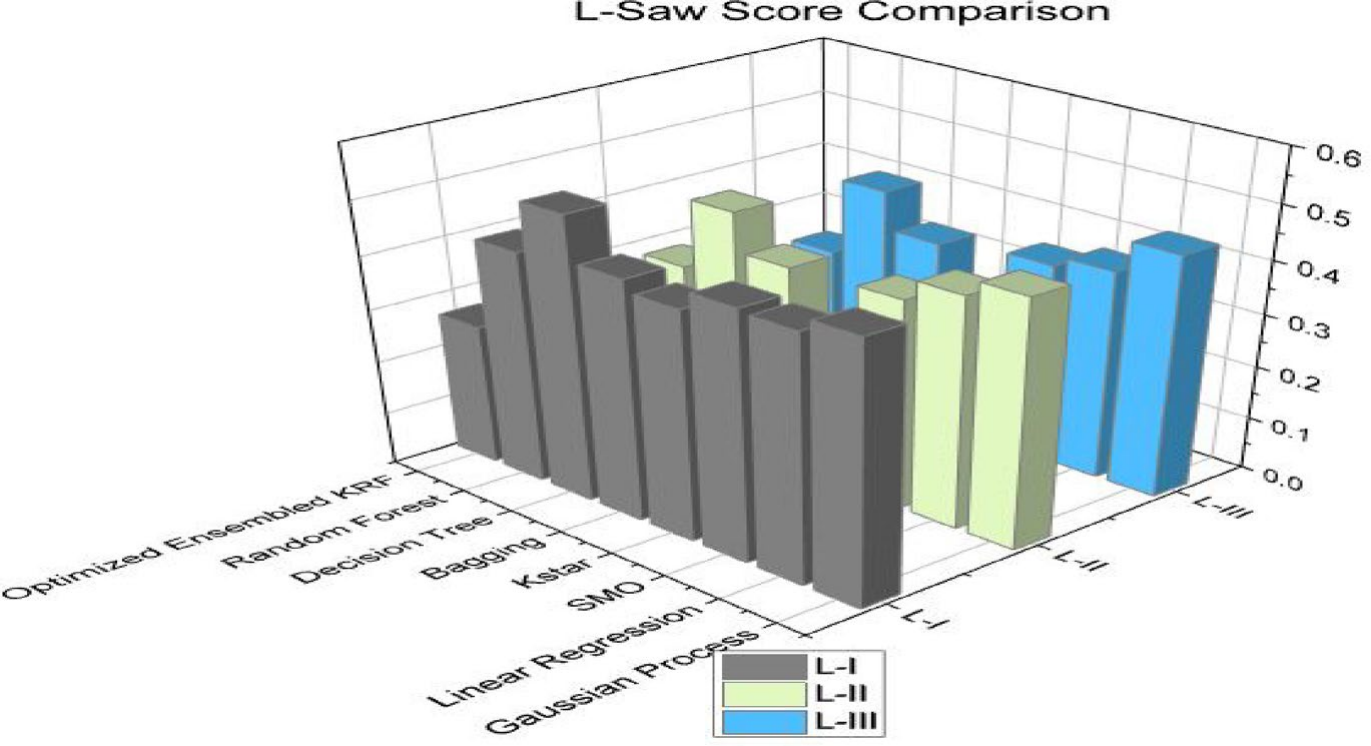
L-saw comparison in all three scenarios.

Further, the concept of W-saw and L-saw scores are introduced to strengthen the optimized toxicity prediction model. The operational research terms W-saw and L-saw are the composite score of multiple performance factors into a single score. W-saw score of the model should be high and L-saw score should be the lowest. Both scores show that the performance of the model is not dependent on the single factor. By pursue these scores leads to monitor changes to the model performance. Tables 12 and 13 represent the W-saw score and L-saw score respectively for different machine learning algorithms. W-saw and L-saw scores comparison is shown separately in Figs. 9 and 10 to present the performance of all models together. The W-saw score for an optimized model in scenario I is 0.83, for scenario II is 0.88, and is best for scenario III by achieving a 0.91 score.

**Table 12:** W-saw score comparison for three scenarios.

Sr. No.	Classifier	W-I	W-II	W-III
1	GP	0.45	0.49	0.53
2	LR	0.51	0.53	0.60
3	SMO	0.50	0.54	0.57
4	Kstar	0.52	0.69	0.78
5	Bagging	0.53	0.60	0.65
6	DT	0.35	0.63	0.66
7	RF	0.55	0.75	0.81
8	OEKRF	0.83	0.88	0.91

The L-saw score for an optimized model in the scenario I is 0.27, in scenario II is 0.20, and is best for scenario III by achieving the lowest value of 0.16.

**Table 13:** L-saw score comparison for three scenarios.

Sr. No.	Classifier	L-I	L-II	L-III
1	GP	0.49	0.47	0.46
2	LR	0.46	0.44	0.40
3	SMO	0.47	0.40	0.38
4	Kstar	0.43	0.28	0.25
5	Bagging	0.45	0.39	0.36
6	DT	0.53	0.46	0.43
7	RF	0.44	0.33	0.28
8	OEKRF	0.27	0.20	0.16

Recent deep learning based models have been introduced and compared with proposed OEKRF model. AIPs-DeepEnC-GA is a deep learning model which combines the strength of deep EnC and genetic algorithm to find the nonlinear relation between molecular structure and toxicity, DeepAIPs-Pred model learns the toxic patterns by monitoring the sequence activities of features and Deepstacked-AVPs model embeds the features and finds all the possible patterns to extend the model generalization for unknown data^42^^–^^43^. Table 14 depicts the performance of deep learning models. AIPs-DeepEnC-GA model performs better with R value as 0.82, COD value as 0.807, MAE as 0.24, RMSE as 0.30 and accuracy of 72%.

**Table 14:** Evaluation parameters for deep learning models.

Classifier	R	COD	MAE	RMSE	Q
AIPs-DeepEnC-GA Model	0.82	0.807	0.24	0.30	72%
DeepAIPs-Pred	0.71	0.5	0.34	0.351	66%
Deepstacked-AVPs	0.62	0.296	0.415	0.419	59%

So our results evaluate that optimized ensembled KRF is best in comparison to seven machine learning algorithms and three deep learning algorithms in all aspects. Table 15 depicts the performance of optimized ensembled KRF in all the three scenarios. The OEKRF model achieved 93% accuracy in Scenario III that shows the strong ability of prediction for assessment of toxicity. Higher values of coefficient of correlation and coefficient of determination makes the model reliable for assessing toxic and non-toxic compounds. Low values of MAE and RMSE make the model compatible for real world applications where prediction of toxicity plays an important role for drug development and predictions at early stage reduces the requirement of extensive testing and saves resources and time.

**Table 15:** OEKRF comparison for three scenarios.

Classifier	R	COD	MAE	RMSE	Accuracy	W-Saw	L-Saw
OEKRF-I	0.90	0.81	0.23	0.3	77%	0.83	0.27
OEKRF-II	0.91	0.83	0.11	0.28	89%	0.88	0.20
OEKRF-III	0.93	0.86	0.07	0.25	93%	0.91	0.16

**Fig. 11:**
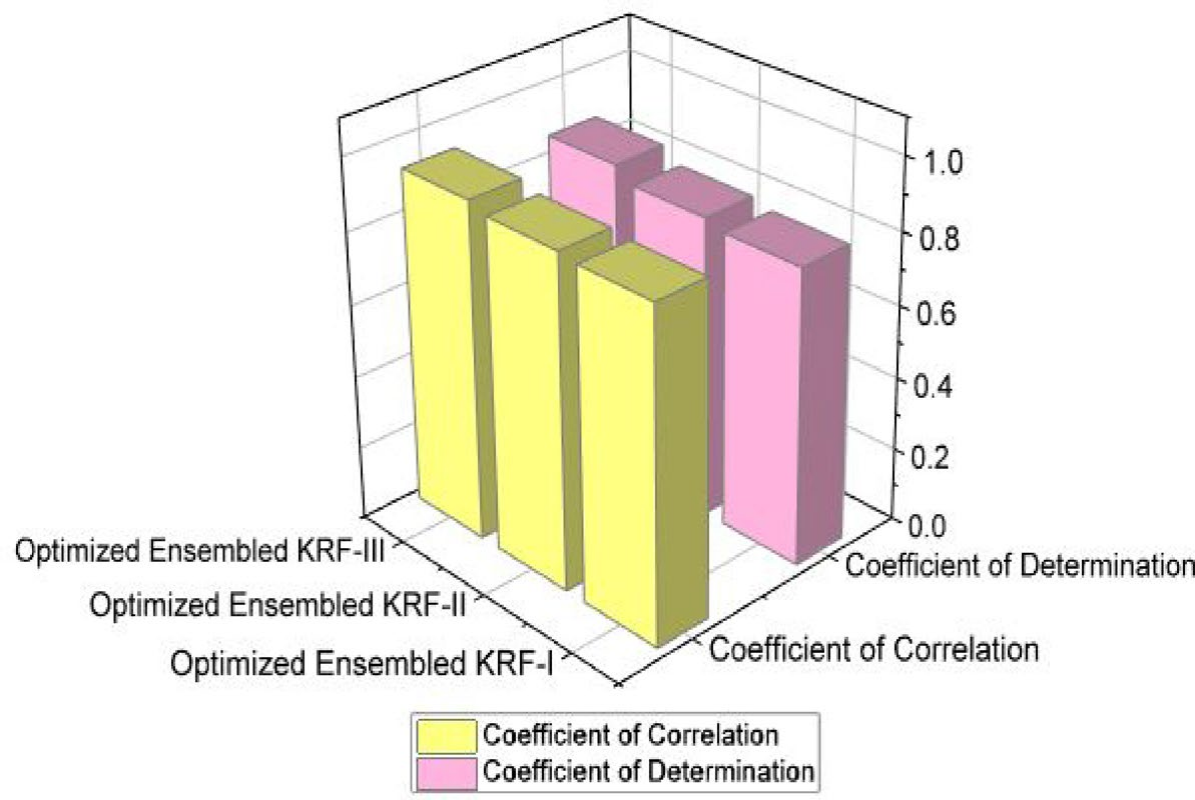
R and COD values comparison in all three scenarios for OEKRF.

**Fig. 12:**
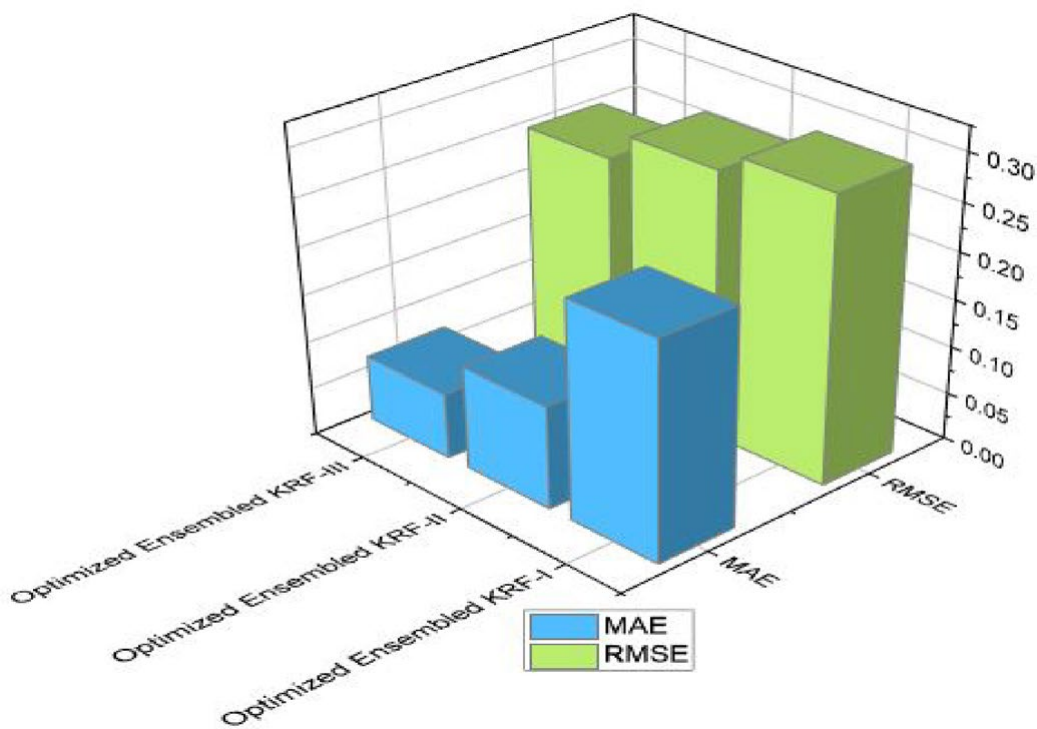
MAE and RMSE comparison in all three scenarios for OEKRF.

**Fig. 13:**
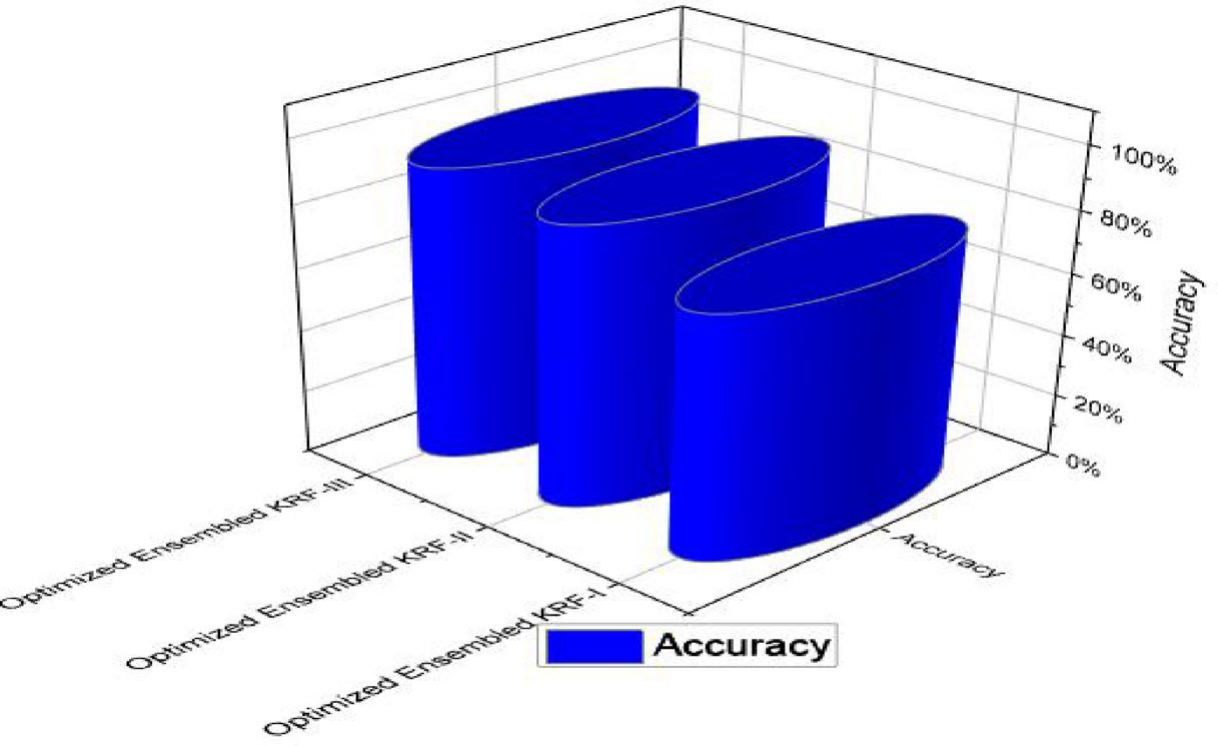
Accuracy comparison in all three scenarios for OEKRF.

**Fig. 14:**
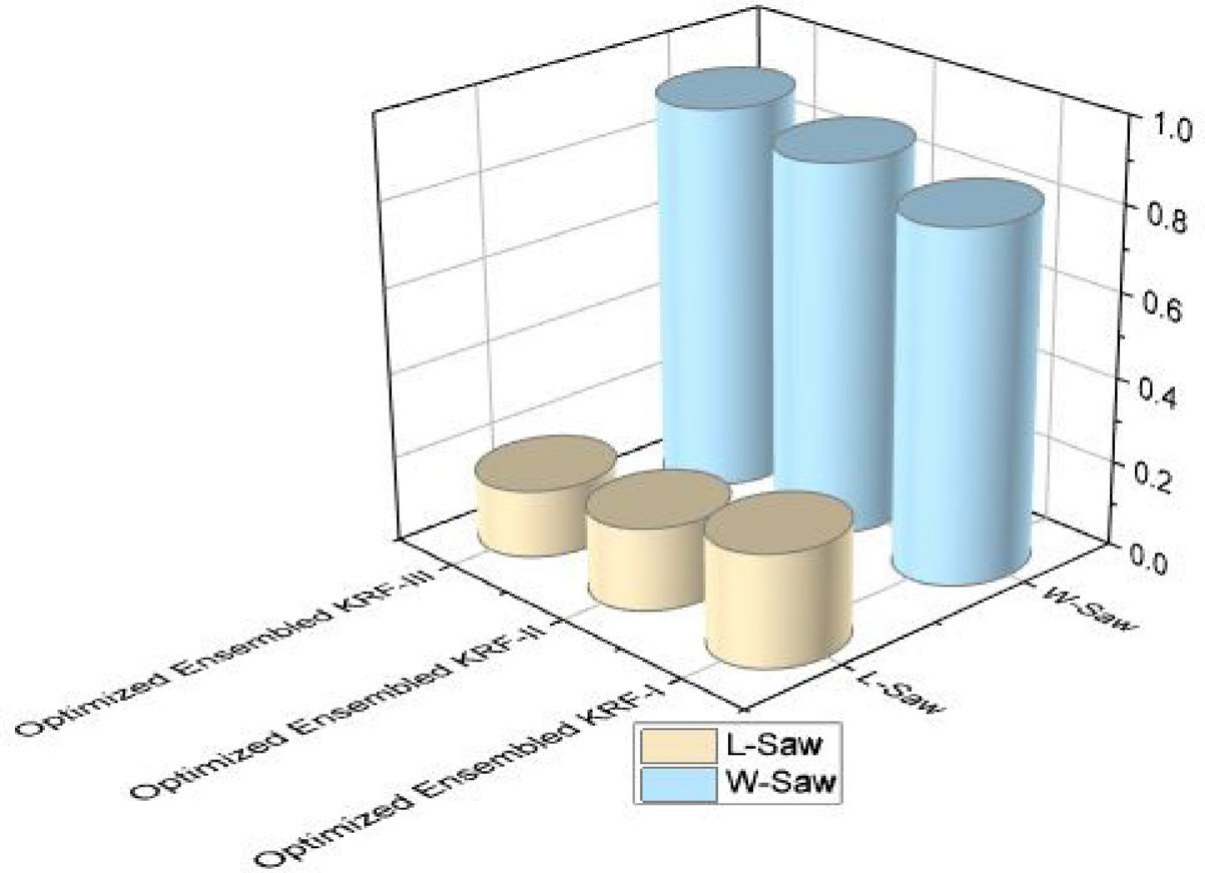
Saw score comparison in all three scenarios for OEKRF.

State of art parameters for all the three scenarios is presented. Figure 11 presents the comparison of R and COD values; Fig. 12 presents comparison of MAE and RMSE values; Fig. 13 is representing accuracy; and Fig. 14 is presenting saw scores with highest and lowest scores.

When the optimized ensembled model KRF itself is compared in three different scenarios, it performs exceptionally well in scenario III with feature selection, resampling, and 10 F-CV.

## Conclusion

Prediction of toxicity has been quite a challenging and crucial task from the start of the medical era. But now AI and ML brought a revolution in the healthcare industry. It is possible to optimize this challenging task now. We developed an optimized ensembled toxicity prediction model KRF in the research. We evaluated and compared state of art parameters for seven machine learning algorithms along with the optimized model. The optimized ensembled model performs well in all three scenarios mentioned in the presented work. Scenario-wise results are shown in the paper with evaluated values of state of art parameters. An optimized model performs exceptionally well in all the scenarios, But when compared to the model itself in all three scenarios, scenario III performs best in all the aspects. Deep learning algorithms are also introduced to compare with the optimized model. The optimized ensembled KRF achieves the highest accuracy of 93% in scenario III which was best in comparison to scenario I and scenario II with values of 77% and 89% respectively. The R value, COD value, MAE value, and RMSE values are 0.93, 0.86, 0.07, and 0.25 respectively for scenario III. Further W-saw and L-saw values for scenario III are 0.91 and 0.16 respectively. So the results are established and validated for all the scenarios but on applying feature selection, resampling, and 10 F-CV technique results are best and optimized. The future prospect of the proposed model (OEKRF) is to easily adopt the sudden changes and works in the dynamic environment and learn from large historical data to identify the patterns which helps to extend the model generalization for unseen data. In the coming era, results can be optimized by using new machine learning algorithms. Optimized combinations of features can be introduced by using new feature selection methods. Ensembling of more algorithms can be performed and results can be analyzed by using new parameters. Although the performance of the model is quite satisfactory but further there is a scope of improvement that leads to use computational science such as automata theory to reduce the computational overhead, recommendation at every level and explore all the possibilities with corresponding solutions.

## Data Availability

Data is available within the manuscript and further can be requested from the corresponding author on reasonable request.
